# Diagnostic challenges of gliosarcoma: case report of a rare glioblastoma histopathological variant

**DOI:** 10.3389/fradi.2025.1687401

**Published:** 2025-10-13

**Authors:** Sergey Karasev, Rustam Talybov, Shamil Chertoyev, Tatyana Trofimova, Vadim Mochalov, Tatyana Kleshchevnikova, Natalya Loginova, Irina Karaseva

**Affiliations:** ^1^ Department of Neurosurgery, Federal Center of Neurosurgery, Tyumen, Russia; ^2^ Radiology Department, Regional Clinical Hospital № 2, Tyumen, Russia; ^3^ Department of Oncology, Radiology and Radiotherapy, Tyumen State Medical University of the Ministry of Healthcare of Russia, Tyumen, Russia; ^4^ Department of Radiology and Radiation Medicine, First Pavlov State Medical University of St. Petersburg, St. Petersburg, Russia

**Keywords:** gliosarcoma, T2WI heterogeneity, glioblastoma, anaplastic meningioma, solitary metastases, multiparametric MRI

## Abstract

**Background:**

According to the 5th revision of World Health Organization (WHO) of central nervous system tumors classification, gliosarcoma is a malignant tumor grade 4 and is the rarest and aggressive subtype of isocitrate dehydrogenase (IDH) wild-type glioblastoma. The special histopathological feature of the tumor is its biphasic differentiation including both the glial and the sarcomatous (mesenchymal) components of the tumor. The characteristics mentioned above create difficulties in radiological and histological diagnoses. Because of its rarity, gliosarcoma is typically not even considered in the differential diagnosis.

**Case presentation:**

This clinical case study describes a 55-year-old man exhibiting acute right-sided hemiparesis and disorientation for 12 h with loss of consciousness. A brain МRI of the patient revealed an intracerebral mass in the left frontoparietal area with close relationship with the dura mater, ring-like enhancement, severe perifocal edema, restricted diffusion of the solid component, internal vascular shunts, microhemorrhages, and elevated perfusion values. At the preoperative stage, the differential diagnosis included glioblastoma, solitary metastasis, and the possibility of an anaplastic meningioma. Tumor microsurgical resection was performed. According to the results of histological and immunohistochemical studies, gliosarcoma was diagnosed.

**Discussion:**

The only characteristic gliosarcoma feature was the phenomenon of solid node heterogeneity detected on the conventional T2-weighted sequence: a combination of hypo- and hyperintense parts. While multiparametric magnetic resonance imaging (MRI) aids in differentiating high-grade gliomas, metastases, and meningiomas, gliosarcoma remains underrecognized because of overlapping features. The observed T2 heterogeneity may serve as a potential radiological marker for gliosarcoma. Accurate and timely identification of brain tumor type is required to establish the appropriate extent of resection in surgical planning.

**Conclusion:**

This case publication does not intend to ignore the data of conventional sequences and instead considers them to be included in the structure of the multiparametric MRI protocol. However, larger studies are needed to validate the findings of this case study and refine diagnostic criteria for this rare tumor.

## Introduction

According to the 5th revision of World Health Organization (WHO) classification of central nervous system (CNS) tumors, gliosarcoma (GS) is a primary malignant tumor grade 4 and is the rare and aggressive subtype of glioblastoma. GS is characterized by aggressive growth, resistance to radiotherapy, and has the worst prognosis (1). The incidence of GS accounts for approximately 2% glioblastomas (2). The median overall survival for patients with GS is approximately 9 months (3–6). Pathology demonstrates the tumor’s biphasic cellular composition of glial and sarcomatous differentiation, posing challenges in establishing a differential diagnosis not only for neuroradiologists but also for pathologists (7). The sarcomatous components are heterogeneous and may include chondral, osseous, osteochondral, myomatous, or lipomatous elements (8, 9). Because of rare incidence, there are limited studies investigating the radiologic features, pathogenesis, diagnosis, or treatment of GS. Existing articles primarily focus on the clinical and histopathological features of the disease. At the same time, specific radiological features remain poorly described. A notable imaging feature is the subcortical location of the tumor, closely adherent to the dura mater (4, 8, 9). Invasion of the skull base and cases of extracranial metastases have also been documented (4, 8, 9). Considering the aforementioned facts, GS is usually not considered in preliminary diagnosis. The primary objective of the surgical management of malignant gliomas is to achieve a gross total resection, which typically extends beyond the margins of the contrast-enhancing portion of the tumor (10). The use of a non-conventional magnetic resonance imaging (MRI) protocol can aid in developing an optimal treatment strategy. In this clinical case study, we aim to demonstrate the radiological features and challenges involved in the differential diagnosis of GS through a specialized multiparametric MRI (mpMRI) protocol.

## Case report

**Figure 1:**
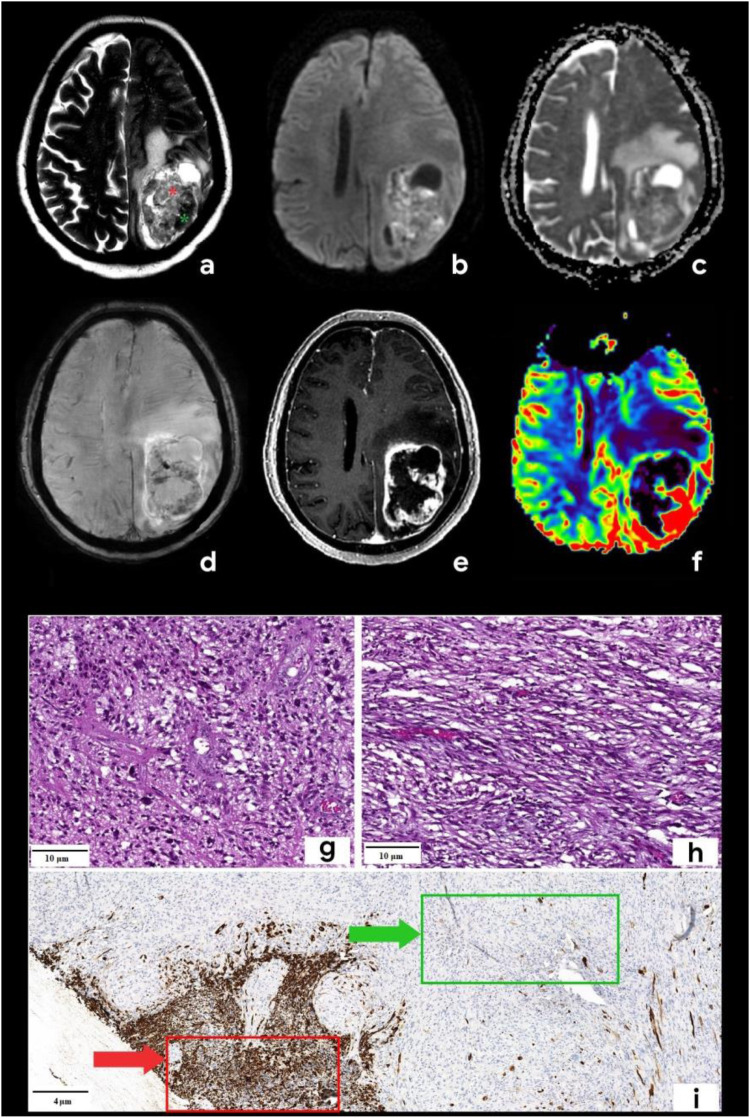
Brain mpMRI and pathology of the patient with gliosarcoma. MRI: (**a**) Т2, (**b**) DWI, (**c**) ADC, (**d**) SWAN, (**e**) T1 + C, and (**f**) CBV. There is a mass with a perifocal edema located in the left frontoparietal area. On T2WI (**a**), the tumor is characterized by signal heterogeneity: a hypointense one (green star) and a hyperintense component (red star). The tumor has a ring-like rim contrast enhancement (**e**), the diffusion restriction of the solid part (**b,c**) with corresponding zones of high CBV (**f**). SWAN (**d**) shows the presence of vascular shunts within the tumor. Pathology: staining scale bar: 4 and 10 µm, hematoxylin and eosin staining (**g,h**) and acidic protein (GFAP), immunohistochemistry (**i**). (**g**) and (**h**) are histopathological signs of classic glioblastoma: the glial part demonstrates the marked nuclear polymorphism and the cellular atypia (**g**), sarcomatous part (**h**) is presented by the spindle-shaped cells with argyrophilic fibers (mesenchymal tissue). I–GFAP demonstrates the marked expression in the glial part of the tumor (red arrow) and the total absence of the expression in the sarcomatous one (green arrow).

A 55-year-old man was admitted to Regional Hospital №2 (Tyumen, Russia) after he experienced a transient loss of consciousness. He presented with complaints of right-sided limb weakness and disorientation for 12 h. Upon admission to the hospital, a computed tomography (CT) scan of the brain without contrast administration was performed using a General Electric Revolution Evo CT scanner (GE Healthcare, USA, Chicago, IL). Axial images were acquired with 0.625 mm slice thickness and reformatted by multiplanar reconstruction (MPR). CT imaging revealed an intra-axial heterogeneous mass in the left frontoparietal region associated with the mass effect, resulting in a brain midline shift. Based on the mpMRI protocol, a specialized radiological examination was performed. A magnetic resonance imaging of the brain was performed using a 1.5-T General Electric Signa Voyager MRI scanner (General Electric HealthCare, China). The mpMRI protocol includes conventional sequences such as T1-weighted imaging (T1WI), T2-weighted imaging (T2WI), T2-FLAIR, and diffusion-weighted imaging (DWI) with apparent diffusion coefficient (ADC) maps. Specialized MRI sequences include sequences sensitive to compounds that distort the local magnetic field [e.g., SWI, susceptibility-weighted imaging (SWAN), T2*] and dynamic susceptibility contrast (DSC) T2-weighted perfusion. Images were acquired in three orthogonal planes (axial, sagittal, and coronal) with a slice thickness of 1–5 mm, both before and after gadolinium-based contrast agent administration. A multiparametric MRI revealed a cystic-solid mass in the left frontoparietal region closely adherent to the dura mater. The lesion was surrounded by a marked area of vasogenic edema and had a severe mass effect. On postcontrast T1-weighted imaging, the tumor exhibited a ring-like enhancement and a vascular network with intratumoral hemorrhages on the SWAN. Perfusion maps demonstrated elevated relative cerebral blood volume (rСBV) within the tumor exceeding normal values of unaffected white matter by three to five times (11). A detailed analysis of the T2-weighted sequence (Figure 1a) revealed signals of heterogeneity within the solid component of the tumor, composed of two distinct regions: a hypointense area (green star) and a hyperintense area (red star).

**Figure 2:**
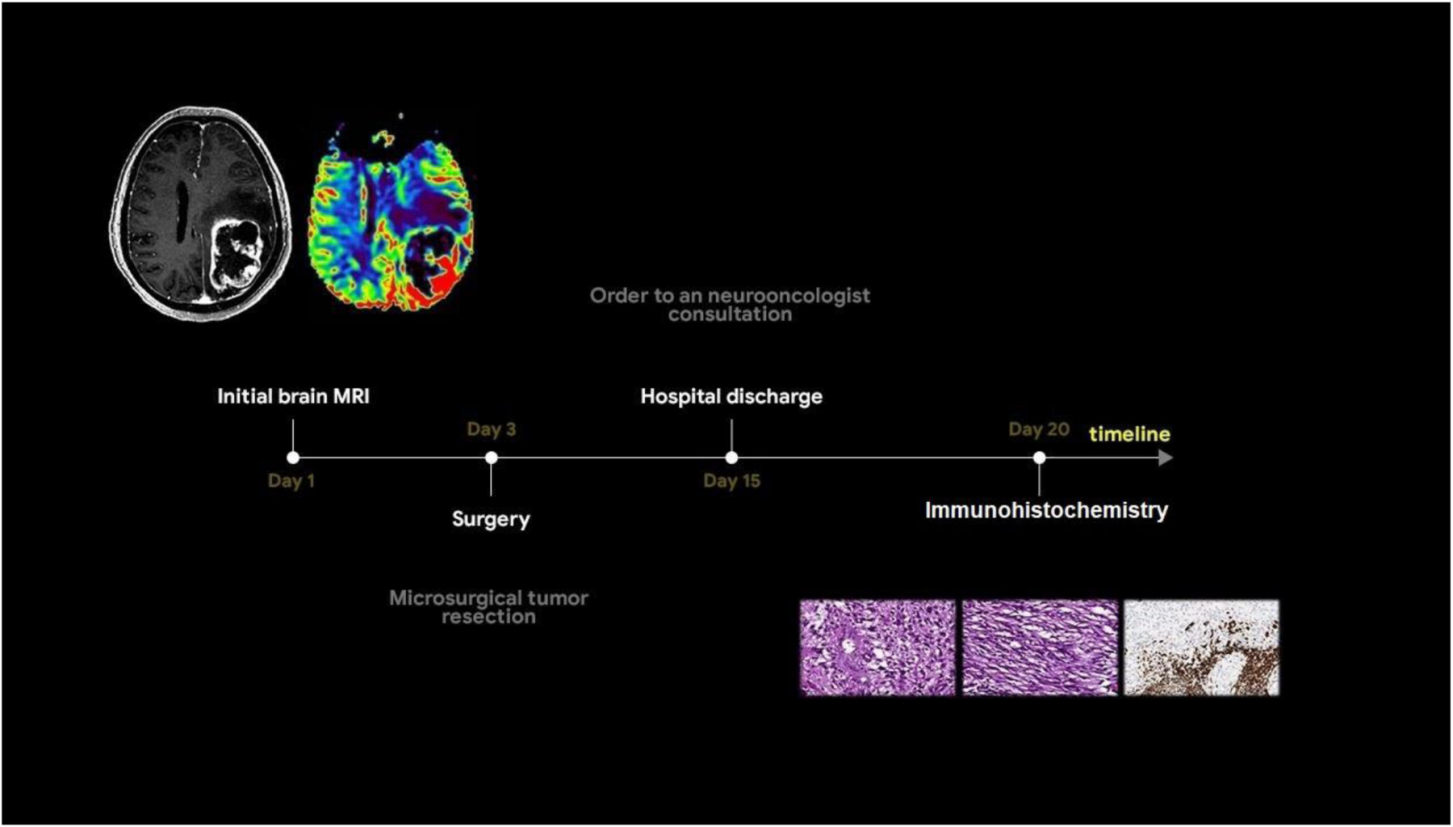
Timeline showing the sequence of events of the case.

Based on the tumor's location and contrast enhancement pattern, the differential diagnosis mainly included solitary metastasis or malignant diffuse glioma but also considered the possibility of anaplastic meningioma. Because of the midline shift syndrome and progressive neurological deterioration posing a life-threatening risk, a microsurgical resection of the mass was performed. The surgical specimen was submitted for a pathomorphological examination. A microscopic analysis identified a rare variant of gliosarcoma characterized by a biphasic pattern: a glial component (Figure 1g) exhibiting marked nuclear polymorphism, cellular atypia, and hyperchromatic nuclei and a sarcomatous component (Figure 1h) composed of spindle-shaped cells arranged in fascicles and interspersed with argyrophilic fibers. An immunohistochemical analysis of the glial fibrillary acidic protein (GFAP) revealed a strong positive expression in the glial component of the tumor (indicated by a red arrow in Figure 1i) and a complete absence of expression in the sarcomatous component (marked by a green arrow in Figure 1i). The patient was discharged in satisfactory condition on the 15th postoperative day and referred to a neuro-oncologist at the Multidisciplinary Clinical Medical Center “Medical city” to determine further treatment strategies and initiate chemoradiotherapy. The diagnostic process of the patient is illustrated in Figure 2.

## Discussion

**Figure 3:**
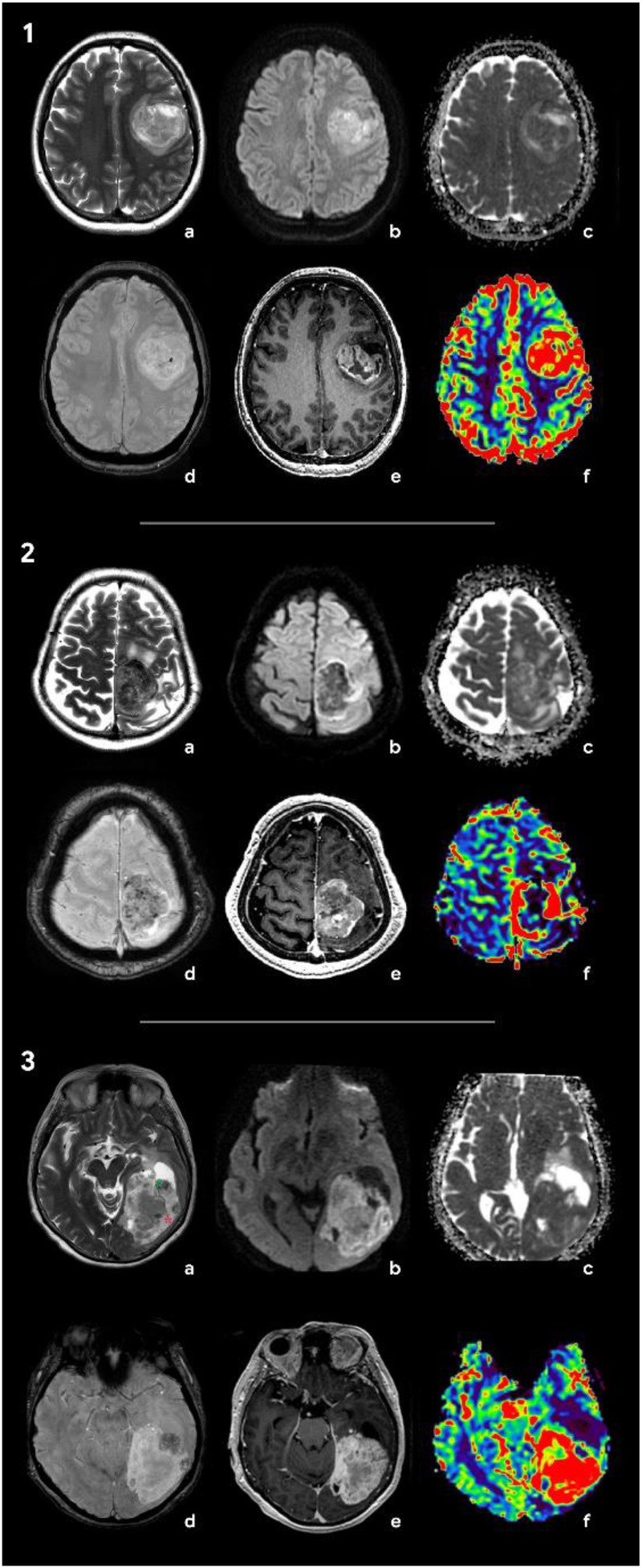
Radiologic features of the tumors included in differential diagnosis: glioblastoma (**1**), solitary metastasis (**2**), and anaplastic meningioma (**3**). (**1**) Brain MRI of a patient with glioblastoma: (a) Т2, (b) DWI, (c) ADC, (d) SWAN, (e) T1 + C, and (f) CBV. In the left frontal area, there is a large axial mass involving the cerebral cortex and white matter. The mass shows a ring-like contrast enhancement and contains necrotic areas. On SWAN, the vascular network and single foci of signal void are marked due to the presence of hemoglobin degradation products. On DWI and ADC maps, diffusion is restricted in the solid tumor component. The CBV map shows an increase in tumor blood flow. Т2 demonstrates a hyperintense signal of the tumor. (**2**) Brain MRI of a patient with brain metastasis of the lung carcinoma: (a) Т2, (b) DWI, (c) ADC, (d) SWAN, (e) T1 + C, and (f) CBV. There is an axial mass located in the left parasagittal region of the junction between the frontal and the parietal lobes. The mass shows a hypointense signal on T2WI and demonstrates vivid contrast enhancement. SWAN detects multiple artifacts caused by microhemorrhages. On DWI, there is a diffusion restriction alongside the tumor periphery. The CBV map shows a threefold increase in tumor blood volume relative to the white matter of the contralateral hemisphere. (**3**) Brain MRI of a patient with anaplastic meningioma: (a) Т2, (b) DWI, (c) ADC, (d) SWAN, (e) T1 + C, and (f) CBV. In the left occipital lobe, an axial mass with a wide dural base is identified. The tumor has a heterogeneous signal on T2WI with hypointense (green star) and hyperintense (red star) components. The tumor shows an intense and homogeneous enhancement and high blood volume values. SWAN depicts no magnetic susceptibility artifacts.

During the preoperative stage, the differential diagnosis included solitary metastasis and high-grade diffuse glioma. There also were suggestions to include in the differential diagnosis anaplastic meningioma. Classic glioblastoma manifestations (Figure 3-1) typically exhibit a ring-like contrast enhancement pattern of the intra-axial solid mass, often accompanied by a central necrotic area. The mass is surrounded by vasogenic edema, in combination with an infiltrative tumor component, which shows no enhancement. Glioblastoma demonstrates a significant mass effect (11). The solid tumor component has restricted diffusion with ADC values measured at 740 ± 200 × 10⁻^6^ mm^2^/s (12). Magnetic susceptibility-weighted imaging reveals signal voids attributable to hemorrhages and vascular shunts. Perfusion studies indicate elevated cerebral blood volume (CBV) levels within both the tumor and the perifocal non-enhancing regions, exceeding unaffected white matter values by over fivefold (11, 13). Notably, conventional T2WI findings, frequently overlooked, could provide critical diagnostic insights. Glioblastomas typically exhibit hyperintense signal intensity on T2-weighted images, reflecting their glial architecture (14). This feature complements advanced imaging modalities in distinguishing glioblastoma from other intracranial pathologies.

Intracerebral metastases (Figure 3-2) are typically characterized by heterogeneous T2-weighted signal intensity, a feature attributable to their variable primary origins and the frequent incidence of hemorrhagic transformation (15). The contrast enhancement pattern varies according to the primary tumor type, with some lesions demonstrating necrotic regions. On DWI, the solid component displays restricted diffusion, with ADC values averaging 867.67 ± 138.6 × 10⁻^6^ mm^2^/s (13). Peritumoral edema is usually non-infiltrative, which can be confirmed by proton magnetic resonance spectroscopy (^1^H-MRS), but is histologically definitive. In addition, metastases lack significant neovascularization, resulting in reduced perfusion parameters (in comparison with glioblastomas and meningiomas) both within the lesion and at the periphery of the enhancing margin (15, 16). Meningiomas typically present with conventional imaging features that facilitate definitive diagnosis during differential evaluation. Most cases demonstrate characteristic dural attachment, although atypical localizations may occur, including intraventricular, epidural, or extracranial sites (16, 17). While the majority exhibit benign histology, atypical and anaplastic variants exist and play both clinical and technical roles; that is, they often mimic aggressive central nervous system malignancies (clinical) and also mimic such malignancies in imaging and histopathological appearances (technical) (16, 17). Malignant transformation in these tumors may manifest carcinomatous, sarcomatous, or melanomatous morphological patterns, with cellular features potentially including rhabdoid or clear cell differentiation (16, 17). Imaging may reveal necrotic areas, cystic degeneration, and hemorrhagic foci. Anaplastic meningiomas generally demonstrate intense homogeneous contrast enhancement, reflecting their hypervascular nature and well-developed vascular network (17, 18). Notably, meningiomas and gliosarcomas share overlapping heterogeneous MR signal characteristics on T2WI, which is attributable to their histopathological composition containing both epithelial-derived (hyperintense) and mesenchymal (hypointense) tissue components (17, 18) (Figure 3-3). This imaging similarity necessitates careful correlation with clinical and histopathological data for accurate differentiation.

The implementation of specialized mpMRI protocols has been conclusively demonstrated to enhance diagnostic accuracy in the differentiation of CNS tumors. However, diagnostic interpretation becomes challenging when tumor nodes are situated adjacent to the corticomedullary junction, particularly in cases lacking definitive white matter involvement (9, 19). In such scenarios, a comprehensive integration of all imaging sequences—including conventional T2WI that may otherwise be overlooked—is critical for accurate assessment. The clinical case of a rare glioblastoma variant exemplifies the diagnostic utility of conventional T2WI, which revealed the intratumoral heterogeneity characteristic of gliosarcoma. This heterogeneity manifested as coexisting regions of hyperintense and hypointense MR signals, a phenomenon attributable to the tumor's biphasic histopathological composition. Specifically, the hyperintense regions correspond to glial components with astrocytic and anaplastic cellular morphology, while hypointense areas reflect densely packed mesenchymal cells. Furthermore, the intermingling of gliomatous and sarcomatous infiltrates creates a distinct irregular pattern characterized by alternating zones of hypointense and hyperintense signals. This imaging signature, arising from the juxtaposition of divergent cellular lineages, is characteristic of gliosarcoma and absent in other CNS neoplasms, thereby underlining its differentiation. Several case series have highlighted one of the typical characteristics of GS—the involvement of the meninges (9, 20). The aforementioned imaging features collectively support the presumptive diagnosis of gliosarcoma during initial diagnostic evaluation, providing critical preoperative data to guide neurosurgeons in optimizing their surgical strategy (21). These radiological findings enable a preoperative delineation of tissue resection boundaries within defined anatomical zones, anticipating the tumor's heterogeneous histological composition during microsurgical intervention. A comparison of the neuroimaging characteristics of the discussed neoplasms is presented in Table 1.

**Table 1:** Comparative characteristics of tumors given in the differential diagnosis.

Сriteria	Gliosarcoma	Glioblastoma	Intracerebral metastasis	Anaplastic meningioma
Localization	Intra-axial, within gray and white matter	Intra-axial, within gray and white matter	Intra-axial, gray-white matter junction	Extra-axial
Involvement of dura mater	Possible	No involvement	Possible	Always
Edema characteristics	Combined: edema + infiltration	Combined: edema + infiltration	Variable vasogenic	Variable vasogenic
Contrast enhancement pattern	Ring-like	Ring-like	Variable	Usually homogeneous, could be ring-like, characteristically intensive
Hemorrhage	Variably	Variably	Often	Rarely
T2WI features	Heterogeneous (alternation of high- and low-signal intensities)	Heterogeneous but without the low-signal intensity	Mainly low-signal intensity	Heterogeneous (alternation of high- and low-signal intensities)
Diffusion-weighted imaging	Restricted in the solid component	Restricted in the solid component	Restricted in the solid component	Restricted in the solid component
Perfusion-weighted imaging	Increased values both within the enhanced and non-enhanced tumor components	Increased values both within the enhanced and non-enhanced tumor components	Increased values but less marked in comparison with diffuse gliomas	Increased values but less marked in comparison with diffuse gliomas and metastases

## Conclusion

Despite overlapping clinical and imaging features among glioblastoma, solitary intracerebral metastasis, and anaplastic meningioma, the multiparametric MRI protocol shows promise in differentiating glioblastoma and its subtypes from other intracranial neoplasms. Further research is needed to fully elucidate their diagnostic potential for rare histological subtypes such as gliosarcoma. The findings presented in this case study suggest that conventional T2-weighted imaging may provide valuable additional insights into tumor heterogeneity, which could aid in preoperative suspicion of gliosarcoma and selection of the appropriate extent of resection during surgical intervention planning. However, the rarity of this tumor variant and the limited number of documented cases highlight the necessity for larger, multicenter studies to validate the observations of this study.

## Data Availability

The raw data supporting the conclusions of this article will be made available by the authors without undue reservation.
